# Brief Report: The Effects of Equine-Assisted Activities on the Social Functioning in Children and Adolescents with Autism Spectrum Disorder

**DOI:** 10.1007/s10803-016-2869-3

**Published:** 2016-07-25

**Authors:** Sophie Anderson, Kerstin Meints

**Affiliations:** School of Psychology, University of Lincoln, Brayford Pool, Lincoln, Lincolnshire LN6 7TS UK

**Keywords:** Autism spectrum disorder, Equine-assisted activities and therapies, Therapeutic riding, Social functioning, Maladaptive behaviour

## Abstract

Equine-assisted activities and therapies are increasing in popularity for treatment of ASD symptoms. This research evaluated effects of a 5-week programme of therapeutic riding on social functioning of children/adolescents (N = 15) with ASD. The effectiveness of the programme was evaluated using the autism spectrum quotient, the Vineland Adaptive Behaviour Scale and the empathising and systemising quotient. Results established that the TR intervention increased empathising and reduced maladaptive behaviours. The findings also indicated that specific adaptive behaviours like socialization and communication were not affected by the intervention. Thus, a complex picture of the effects of this intervention emerges: while TR does not change all of the child’s behaviour, it can improve specific aspects of social functioning and also reduce maladaptive ASD traits.

## Introduction

Historically, the ancient Greeks regarded horseback riding not just as a means of transportation, but as a way of improving health and well-being for the disabled (Hallberg 2008). Hippocrates was the first to describe the benefits of horses for rehabilitation purposes, calling horseback riding ‘universal exercise’ and the word ‘hippotherapy’ originates from ancient Greek as “treatment with the help of a horse” (Hardy 2011). The term Equine-assisted activities and therapies (EAAT) was developed in the 1990s, although these activities have been gaining in popularity for decades (Kersten and Thomas 2005).

**Fig. 1:**
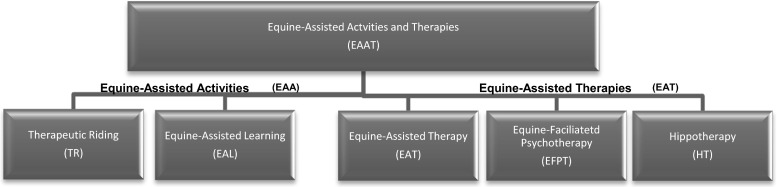
Overview of definitions of Equine-assisted activities and therapies (EAAT) according to PATH (2016)

There is little uniformity across professional bodies regarding the terminology for describing EAAT. For clarity, this research will use the terms defined by the Professional Association of Therapeutic Horsemanship International (2016), definitions of the EAAT can be found on the PATH website (2016), see Fig. 1 below for an overview.

According to PATH definitions, therapeutic riding (TR) is an equine-assisted activity designed for the purpose of contributing positively to the cognitive, physical, emotional and social well-being of individuals with special needs (Ward et al. 2013). TR emphasises attention, control, focus, sensory management as well as nonverbal and verbal communication in order to teach riding skills (Fine 2010, 2015; see also Gabriels et al. 2015 for an overview).

The different types of EAATs are conducted by professionals in the relevant fields and utilise the horse’s natural instincts to promote behavioural changes (Maclean 2011). Recent attention has focused on the use of EAAT as a viable option for a variety of developmental disorders, including autism spectrum disorder (ASD) (Kern et al. 2011).

ASD is a developmental disorder, characterized by problems with verbal and non-verbal communication, social integration and awareness, stereotyped repetitive behaviours or interests as well as deficits in motor skill functioning (Zonneveld et al. 2012). The prevalence of ASD has risen nearly 600 % in the past two decades, with estimates suggesting that 1 in 68 children worldwide have a clinical diagnosis (Centres for Disease Control and Prevention 2014). Whilst this rise may be due to improved diagnostics or better understanding of the condition, ASD’s exact aetiology is still unknown. The onset of ASD generally appears before 3 years of age (Autism Society of America 2009), but due to the fact that ASD is highly complex and heterogeneous in nature, it has proven difficult to operationalize a single measure to fully assess its multidimensional aspects (Rajendran and Mitchell 2007). Implementation of appropriate therapies and interventions has proven to be beneficial, however, treatment of social function deficits still remains one of the most challenging areas (Scattone et al. 2006). Social functioning has been defined as one’s ability to construct representations of relations between oneself and others, and to use such representations to model social behaviour (Adolphs 2001).

Current research has indicated that TR can be a suitable modality for intervention for those with a spectrum disorder (Umbarger 2007). Horses have been described as detecting minute changes in a human’s body language, thus providing a ‘mirror’ for the participant to gain insight into their own psyche (Schultz et al. 2007; see also McCormick and McCormick 1997). Likewise at the physiological level, behavioural and evolutionary biology has indicated that there are basic universal mechanisms and structures underlying both social behaviour in humans and animals, enabling interspecies social relationships to develop, thus affecting human social behaviour (Beetz et al. 2011). Research by Kern et al. (2011) reported improvements in the severity of symptoms commonly associated with ASD, based on 20 participants completing a 6-month TR program. The Childhood Autism Rating Scale (CARS) was used for evaluation alongside standardized behavioural and physical assessments, demonstrating positive benefits. Similarly, Bass et al. (2009) looked at the effects of a 12-week TR programme on social functioning in children (*n* = 34) with ASD, the results showed an improvement in sensory integration, directed attention and social motivation.

Gabriels et al. (2015) research expanded on their 2012 pilot study with the research focusing on communication, self-regulation, socialization, adaptive and motor behaviours The research provided the first randomised, large-scale (*n* = 127), controlled trial to demonstrate the efficacy of TR for the Autism population.

Research has also shown that TR encourages children to work with their hands, exercise and connect with the horse, as well as with those implementing TR (Dingman 2008). Contradictory to the majority of TR findings are those of the experimental study by Jenkins and Reed (2013) who suggested no clinically significant effects for improvement of ASD behaviours including language, compliance and problem behaviour (Jenkins and Reed 2013). However, a multiple case design by Holm et al. (2013) challenged these results, indicating a significant magnitude of change (70 % from the baseline) in targeted behaviours including spontaneous communication in children with Autism. Using the CARS Aberrant Behaviour Checklist alongside direct observation, the study examined the dosage and generalization of the benefits of TR to home and community. Findings indicated that increased ‘dosages’ of TR led to better retention.

In a recent paper, Borgi et al. (2016) tested 28 children with ASD once a week for about 60–70 min over a 6-month period, not within a TR, but within an EAT programme and found improvement in social functioning, improved executive functioning and a weaker effect on motor abilities at the end of the programme.

The current study builds on existing EAAT research by systematically examining the effects of TR and horsemanship skills on social functioning in children and adolescents with ASD. The purpose of this study was to build upon the limited data available on the effectiveness of TR, focusing on aspects of social functioning and maladaptive behavioural traits while measuring these with standardised instruments before and after intervention. In addition, as EAAT is often used in practice without being measured systematically, we aimed to undertake first steps to measure existing practice in a current setting.

## Method

### Participants

The participants were attained from Saint Nicholas Academy for Autism Trust (SNAAP), charity registration number 1104306. The SNAAP charity uses “The Stables Riding for the Disabled and Activities Centre” to carry out EAAT programs during the school holidays. The SNAAP summer programme consisted of EAA: EAL and TR sessions. The researcher carried out initial screening with ‘The Stables’ manager to find the most suitable candidates for the research from the wide range of clients who use their services. Participant screening was conducted after SNAAP was identified and agreed to participate in the research. Some SNAAP service users, although SNAAP is primarily for Autism support, did not have a diagnosis of Autism or Aspergers, so were not accepted as candidates for the research. Candidates were also required to have had no riding experience with horses before. While it was not an exclusion criterion for participants to be verbal or non-verbal, all participants in the sample had limited verbal ability. No other exclusion criteria were used. Parents were asked if their children needed any additional learning support, use of Makaton or pictorial support, which some members of staff were trained in, but this was declined.

The participants (*N* = 15) were aged between 5 and 16 years (*M* = 10, *SD* = 3.8) with a current clinical diagnosis of ASD. All participants were clinically diagnosed by health care professional (using DSM) and were all registered as disabled (see Borgi et al. 2016 for similar inclusion procedure). The sample comprised of 11 males and 4 females, corresponding to the gender difference in the ASD population of 4:1 male to female (Baron-Cohen and Wheelwright 2004). As typical for ASD, some of the sample had comorbid disorders, including Attention Deficit Hyperactivity Disorder (ADHD, 20 %), Hypersensitivity and Sensory Integration Disorder (53 %) (Autism Society of America 2009). The sample consisted of 27 % of participants with ASD, 20 % with ASD and ADHD and 53 % with ASD and related conditions.

Parents were emailed consent forms previous to the start date and asked to complete them and bring them to the initial assessment day at ‘The Stables’.

### Measures

A mix of parent-report questionnaires and semi-structured tests was administered on the first and last day of the EAA intervention, the Autism-Spectrum Quotient for Children (Auyeng et al. 2008) and the Autism-Spectrum Quotient for Adolescents (Baron-Cohen et al. 2006) were chosen as the ASQ has been shown to be successful in capturing some core dimensions of individual differences and giving an understanding of ASD traits in individuals (while not being a diagnostic tool). Two variations of the ASQ were used due to the age range (5–16). The ASQ-Child was administered for 4–11-year-olds and the ASQ-Adolescents was used for participants aged 12–15. The questionnaires were completed by the parent/carer, which took approximately 20–40 min. The scores between the ASQ-Child and ASQ-Adolescent were transformed into percentages to allow a comparable analysis.

The empathising quotient/systemising quotient (Auyeng et al. 2009) is a 55-item Likert type parent-report questionnaire to evaluate participants on the skills underlining the empathising–systemising (E–S) theory (Baron-Cohen 2009; Baron-Cohen and Wheelwright 2004; Baron-Cohen et al. 2003). The questionnaire took approximately 15–30 min to fill out. Answers were used to provide an EQ score out of 54 and SQ score out of 56, with higher scores relating to more empathising or systemising traits respectively. The difference between the two scores was measured to calculate the EQ/SQ score. Emphasis on limited empathising explains why ASD sufferers have social difficulties while increased systemising leads them to have a need for control, repetitive behaviours and narrow interests (Baron-Cohen 2009).

The Vineland Adaptive Behaviour Scale (VABS) (Sparrow et al. 1984) was used as it measures subdomains of adaptive (socialization, communication, daily living skills, motor skills) and maladaptive behaviour. The maladaptive behaviour section of the VABS, focuses on behaviours which inhibit a person’s ability to adjust to particular situations. These include internalized, asocial and externalized behaviours, such as destructive habits, repetitive behaviours, self-harm tendencies. The VABS takes approximately 30–60 min to complete with the parent/carer by the researcher.

### Procedure

Prior to the study, ethical consent for the study was obtained from the University of Lincoln, School of Psychology Ethics committee. The researcher visited the horse centre prior to the research to ensure suitable equine welfare and standards of practise took place. The Stables Horse Activity Centre, Registered Charity 1108451 has been running for over 20 years working specifically with children and adolescents with disabilities and special needs to offer TR, EAL and Hippotherapy. When the researcher visited the site a particular service user demographic was articulated. In order to meet the requirements for inclusion in the research, a current ASD diagnosis was required, undertaken by certified Healthcare professionals and no prior experience of horse riding.

Informed consent was received from all participating in the study. All participants were briefed and debriefed in full before and after sessions by the researcher.

The participants attended for a total of 6-weeks EAA, with an initial assessment day, followed by the 5-week programme of one 3-h session per week. The initial assessment day was for the staff of ‘The Stables’ to understand the children’s needs and to develop a safe structure for the ensuring programming enabling maximised learning from all participants. The researcher stayed blind to the goals. Parents and carers were not participating in the programme itself. The stable manager carried out risk assessments to see how many members of staff/volunteers were needed to safely carry out the EAA with each participant and how big the groups could be.

Staff at ‘The Stables’ are qualified within the equine industry as instructors with the British Horse Society (BHS) and Riding for the Disabled (RDA) and specially trained to work with children with disabilities and special needs. Participants and parents/carers were required to complete ‘The Stables’ liability and Health and Safety forms.

#### The Programme for the Children

The Stables carried out an initial full day (8 h) for individual assessment including risk assessments for each child. In week 1, an initial health and safety briefing was carried out by members of The Stables staff. Parents completed self-assessments and interviews with the researcher after a brief introduction. The child participants did horsemanship activities including grooming, leading and mucking out throughout the course of the day with plenty of breaks to allow the participants to relax and get involved. Individual TR lessons were conducted to assess the level of need for support and were carried out by an RDA instructor. Time spent doing individual tasks was not specified and varied based on the participant’s engagement and ability.

After this first week, there was one 3 h session per week. Sessions were tailored to each individual based upon goals determined by the initial assessment. They carried the same format each week and were conducted by a trained TR instructor and experienced volunteers. Volunteers had to meet ‘The Stables’ criteria for proficiency of horsemanship skills, some of whom were disabled and had previously been clients. Participants were split into 3 groups (*N* = 5). Activities and exercises that addressed physical, psychological, cognitive, and social skills were incorporated into the program, which was split into 3-subsections; TR, Horsemanship and Stable management. Each group did an hour of each type of activity in varying orders. The routine for each type of activity remained consistent and was carried out by The Stables staff:

#### Therapeutic Riding

Tack up, lead to arena, mount, ride, dis-mount, lead horse to stable, untack.

#### Horsemanship

Head collar on horse in stable, tie up horse on the yard, identify grooming brushes, groom with different brushes, hoof pick, lead horse, return horse to stable.

#### Stable Management

Take out feed and water buckets, get tools (wheel barrow, skip bucket, plastic fork, broom and shovel), clean stable, empty wheelbarrow, fill water buckets, make feeds and hay.

Questionnaires for the parents were administered by the researcher before and after intervention: Each parent/carer was given the autism spectrum quotient and the empathising/systemising quotient to complete pre- and post-intervention. The researcher conducted the Vineland Adaptive Behaviour Scales with each parent in time slots allocated pre- and post-intervention. The researcher has been trained in interview techniques for the use of the tests. A full de-brief with parents and participants was conducted after the last session.

### Data Analysis

The data gathered was analysed using statistical analysis, including ANOVAs with before and after measures on each of the tests as within-subjects factor, and Age group (younger/older) and Caregiver (caregiver/parent) as between-subjects factors. All statistical procedures were performed using SPSS 19. Statistical significance was set at *p* < 0.05. When significant differences were found, comparisons were performed using *t* test with Bonferroni correction.

## Results

Descriptive analysis on the data show the mean (*M*) and standard deviation (*SD*) scores for each of the measures as well as the changes in scores after TR, see Table 1 below.

**Table 1:** Mean and SD across all measures

Measure	Pre-EAT		Post-EAT	
	*M*	*SD*	*M*	*SD*
Autism spectrum quotient (ASQ) percentage score*	62.13	24.20	60.66	24.19
Vineland Adaptive Behaviour Scale** (VABS)	210.13	124.32	214.86	121.63
VABS communicative domain	44.20	31.19	44.60	30.39
VABS socialization domain	45.27	23.21	45.07	22.48
VABS maladaptive behaviour	28.26	12.82	26.73	12.41
Empathising quotient (EQ)	14.86	10.21	16.20	9.32
Systemising quotient (SQ)	26.53	14.20	26.27	13.72
EQ/SQ	14.86	16.73	13.93	14.99

* The percentage was calculated to compare ASQ-Child and ASQ-Adolescent scores

** The adaptive behaviour composite of standardised scores for the VABS

To investigate which of these differences pre- to post- intervention were statistically significant, we calculated a series of ANOVAs for the different measures.

### Autism Spectrum Quotient

The ASQ scores were examined with an ANOVA of Age Group (under 11; 11–16) × Caregiver (same, different caregiver) × EAA (pre, post). A highly significant main effect of EAA was revealed [*F*(1,10) = 11.195, *p* = 0.007, η_p_^2^ = 0.528] with a clear reduction in ASD traits being indicated post-EAA (see below or detailed analysis). There were no main effects of age or Caregiver on ASQ scores. However, the interaction of caregiver on pre- and post-ASQ scores reached significance [*F*(1,10) = 5.934, *p* = 0.035, η_p_^2^ = 0.372]—this may indicate that having the same caregiver or a different parent/carer fill in the forms pre- and post-intervention affected the ASQ results. Further analysis was conducted using a pairwise *t*-test with Bonferroni correction (*p* value of 0.025), however, this showed no significant difference for the same versus other caregiver completing the ASQ pre- and post-EAA *t*(8) = 1.000, *p* = 0.347. There were no other interaction effects. Thus, we can conclude that overall effects of caregiver identity do not hold statistical significance.

### Vineland Adaptive Behaviour Scale

The VABS adaptive behaviour composite scores were examined with a 3-way ANOVA of Age group (under 11, 11–16) × Caregiver (same, different) × EAA (pre, post). This ANOVA just misses significance on the VABS scores *F*(1,11) = 4.772, *p* = 0.051, η_p_^2^ = 0.303 suggesting that the VABS adaptive score did not significantly improve due to the EAA. There were no other main effects or interactions for the VABS adaptive behaviour composite.

As age and caregiver did not affect the results of the VABS pre- and post-EAA, they were not factored into analysis for the individual subdomains of communicative or socialization. A *t* test was conducted on the VABS Communicative domain scores pre- and post-EAA, however, there was no significant difference [*t*(14) = −0.792, *p* = 0.442] demonstrating that communication as measured by the VABS did not improve as a results of the EAA. A further *t* test also indicated that there was no significant difference in scores in the VABS Socialization domain [*t*(14) = 0.564, *p* = 0.582] showing that socialization did not improve due to the EAA.

### Maladaptive Behaviour

The maladaptive Behaviour scores were examined with a 3-way ANOVA of Age group (under 11, 11–16) × Caregiver (same, different) × EAA (pre, post). This revealed a significant main effect of EAA on the VABS maladaptive behaviour score [*F*(1,11) = 5.65, *p* = 0.037, η_p_^2^ = 0.339] indicating a reduction in maladaptive behaviour traits after EAA. There were no other main effects or interactions for the VABS maladaptive behaviour domain.

### Empathising and Systemising Quotient

The Empathising scores were examined with a 3-way ANOVA of Age group (under 11, 11–16) × Caregiver (same, different) × EAA (pre, post) This analysis showed a significant main effect of the EAA on the EQ score [*F*(1,11) = 5.19, *p* = 0.04, η_p_^2^ = 0.320] demonstrating that there was an improvement in empathising due to the EAA. No other main effects or interactions reached significance for the EQ.

Another 3-way ANOVA was carried out to examine the effects of EAA on systemising scores (SQ) with Age group (under 11, 11–16) × Caregiver (same, different) × EAA (pre, post). The ANOVA demonstrated that there was no main effect of EAA on the SQ score [*F*(1,11) = 0.56, *p* = 0.470, η_p_^2^ = 0.048], thus indicating that there was no improvement in systemising after EAA. No other main effects or interactions reached significance for the SQ.

A *t* test was also conducted to look at the difference between the EQ/SQ score pre- and post-EAA. There was no significant difference between the EQ/SQ score pre- and post-EAAT [*t*(14) = 0.940, *p* = 0.363], thus demonstrating that the empathising and systemising quotient did not show any changes due to EAA.

## Discussion

The results of this study suggest that EAA may have an effect on improving aspects of social functioning in children and adolescents with ASD. Findings demonstrated that there was a positive reduction in maladaptive behaviour traits and an improvement in empathising. However, EAA did not demonstrate significant improvement in overall adaptive behaviours, more specifically, there were no significant improvements in communication and socialization.

The significant improvements in maladaptive behaviours, including internalised, asocial and external behaviours have countless benefits and implications for the day-to-day life of a child or adolescent with ASD (Sparrow et al. 1984). Beetz et al. (2011) proposed that this includes a positive modulatory effect on stress, trust and psychological health, enabling improved social functioning through increased physical closeness, contact and non-verbal communication. Direct physical contact with the horse is seen as the key factor for promoting positive behavioural change (Beetz et al. 2011). Bass et al. (2009) support this notion, suggesting that the physical presence of the horse and its rhythmic movement add to the multisensory nature of the TR benefitting social functioning. Zonneveld et al. (2012) propose that this is due to children forming relationships with animals which subsequently enables the children to form bonds with people, leading to a reduction in maladaptive behaviours. These findings resonate with Kern et al. (2011) who reported improvements in the severity of symptoms commonly associated with ASD following a 6-month TR programme. Interestingly, Hawkins et al.’s (2014) results indicate that improved social functioning may also be due to the improvements in motor skills gained by participating in EAT which may consequently enable the development of age-appropriate social skills.

Research by Volkmar et al. (1987) documented that whilst deficits in social factors are important defining characteristics of ASD, early research had not systematically evaluated social dysfunction, including maladaptive traits. The findings of this research suggest that social dysfunction can be targeted through EAA, reducing negative traits shown in an individual, even if adaptive behaviours did not benefit in this instance. Positive improvements in communicative behaviours and socialisation as measured by the VABS were not found in our research, contrary to findings that expressive language, eye contact and mood improved due to TR (King 2007).

Gabriels et al. (2015) who implemented a 10-week TR program and used next to the VABS also the Peabody Picture Vocabulary Test (PPVT-IV) also found significant improvements in spoken language and use of new words. The Peabody Picture Vocabulary Test (PPVT-IV) may have been a useful addition to our current test battery and future studies should incorporate more specific language and communication tests if assessing improvements in these areas.

Due to the multisensory nature of the various forms of EAAT overall, it is hard to pinpoint what the defining factor in promoting positive change is. Bass et al. (2009) substantiate that the act of riding being seen as a rewarding stimulus could account for higher motivational levels and social engagement. These findings contradict those of Jenkins and Reed (2013) who suggested that TR did not produce clinically significant improvement of ASD behaviours including language, compliance and problem behaviours in children (albeit with a small sample). This is congruent with the results of the VABS indicating there was not an improvement in communication or socialization.

Results of this research also indicated decreased ASQ scores and an improvement in ASD traits; including a lack of social awareness, integration or motivation, problematic verbal and non-verbal communication (Zonneveld et al. 2012). Improvements in these key areas correlate with Bass et al. (2009) who reported improvements in social motivation and sensory attention.

Our EQ results show clear improvements in empathising. This further supports findings by Bizub et al. (2003) whose evaluation of a TR program demonstrated increased psychosocial benefits, including empathizing. Baron-Cohen (2009) states that a deficit in empathy causes social difficulties. This is consistent with Vandereijcken et al.’s (2008) perspective that deficits in social interactions pertain to ASD sufferers lack of understanding of others.

EAA in our study may have been successful for improving empathising as Nebbe (2003) suggests it is easier to teach children to be empathetic with animals, since children see animals as peers. Likewise, Ewing et al. (2007) found that empathy could be aided during a child’s social maturation through interaction with animals. Improving empathy in children with ASD aids social functioning by increasing one’s ability to analyse and identify emotional states in others and respond suitably (Sowa and Meulenbroek 2012).

Whilst humans communicate verbally and non-verbally through eye contact and facial expressions, the success of EAA or EAAT more generally may lie in the fact that animals make their behavioural intentions clearer non-verbally (Prothmann et al. 2009). Not mixing verbal and non-verbal communication to transmit emotion-related information may make horses more logical to people with ASD than humans (Prothmann et al. 2009).

Likewise Gabriels et al. (2012, 2015) demonstrated that TR is beneficial for facilitating these improvements due to the horse’s behavioural response giving direct feedback to the child, enabling them to gain a better social understanding and become more self-aware. Improved empathising may therefore pertain to a variety of factors including increased control of non-verbal communication and body language needed to command respect, gain trust and build a bond with horses (Pendry and Roeter 2013).

Whilst the research indicated an improvement in empathising, it did not demonstrate a decrease in systemising. Systemising is a dominant trait within the ASD population (Zonneveld et al. 2012) displayed in stereotyped repetitive behaviours interests and need for control which is largely but not completely independent of empathising (Baron-Cohen 2009). There are a variety of factors that could be attributed to the lack of improvement, however, as systemising is generally typical or superior in those with ASD, and as we see increases in empathising with EAA, programmes might focus on improving social functioning through increasing participants empathising (Wheelwright et al. 2006).

Whilst this research yielded results supporting the benefits of EAA, there are also clear limitations of the study. The most notable limitation is the small sample size. As we aimed to keep the research as non-intrusive as possible and aimed to measure existing practice, we were bound by the natural group size within the existing groups. Ideally a matched control group participating in another alterative therapy e.g. Art or Dance therapy would have been used for comparison of EAA effectiveness instead of before and after measures only. However, due to time and monetary constraints of this study, this was not possible—however, as the results point at a differentiated picture of effects, our results will be interesting to follow up by further research.

Another potential criticism of the study could be the caregiver completion of the self-assessment forms and interviews. Despite results showing no significant differences between different carers, one might argue that it may be an advantage to have the same parent/caregiver completing pre- and post- evaluation forms. However, in real-life circumstances which our research reflects, this may not always be possible as shared care-giving for children with special needs is common.

Regarding the contents of the EAA, Jenkins and Reed (2013) noted that the content of each lesson is a key factor to the success of EAA. Beetz et al. (2011) also suggest that the quality of the therapeutic relationship has been identified as the key factor for success. It may therefore be useful for future work to also evaluate the intensity of any EAAT and the standard of the service offered.

Considering the varying length of EAAT or EAA programmes, generally ranging from 2.5 h to 6-months (Jenkins and Reed 2013), this study implies that a 5-week EAA program can be effective for improving aspects of social functioning when sessions are directly tailored to each individual’s needs. This notion is supported by Gabriels et al. (2015) research—demonstrating improvements in the TR group after 5-weeks, showing reducing irritability, and hyperactivity compared to the control group. Zonneveld et al. (2012) suggested a care-home approach, which may provide an alternative with sessions on a daily basis enabling continuity and working with traits such as the need for repetition. This is not always practical or suitable, but an increased concentration of sessions would likely benefit participants, for example, if more time was available, such as in the school holidays. Ideally, longitudinal research should be carried out to assess the effectiveness of EAAT programmes over time and to see if improvements are sustained.

For future studies, observations of interactions between child and horse-practitioner could also be beneficial to investigate further aspects of effective promotion of improved social functioning.

In general it is proposed that EAAT practitioners use standardised measures and tools to regularly assess their program’s effectiveness, in order to evaluate what is being targeted and to maintain a high standard of practise.

We conclude that EAA and more broadly, EAAT is a useful therapeutic option for improving social functioning in children and adolescents with ASD, as long as it is well-designed and implemented. EAAT research and the current findings also suggest that using horses to alleviate ASD symptoms in children does not alleviate all symptoms, but some and does indeed contribute to their general well-being and psychological health.
